# Neurological Masquerades of Diffuse Large B-cell Lymphoma: A Case Mimicking Motor Neurone Disease

**DOI:** 10.7759/cureus.118661

**Published:** 2026-10-27

**Authors:** Ibnul Shams, Rayhanul I Tuhin, Habib Ullah Abir, Md Romman Khan

**Affiliations:** 1 Haematology, United Lincolnshire Hospitals NHS Trust, Boston, GBR; 2 Oncology, United Lincolnshire Hospitals NHS Trust, Boston, GBR; 3 Haematology, Pilgrim Hospital, Boston, GBR; 4 Internal Medicine, Pilgrim Hospital, Boston, GBR

**Keywords:** anterior horn cell disorder, diffuse large b-cell lymphoma, electromyography, motor neurone disease mimic, non-hodgkin lymphoma, paraneoplastic neurological syndrome

## Abstract

Diffuse large B-cell lymphoma (DLBCL) is the most common subtype of non-Hodgkin lymphoma (NHL) and typically presents with lymphadenopathy, constitutional symptoms, or extranodal disease. Neurological presentations are uncommon and may mimic primary neurological disorders, which creates significant diagnostic challenges.

A 67-year-old woman presented with progressive lower back pain, paraesthesia affecting all four limbs that left her bed-bound, faecal incontinence, dysphagia, weight loss, and evolving neurological deficits, including left-sided facial weakness and asymmetric tetraparesis predominantly affecting the left side. Initial neuroimaging demonstrated no acute intracranial pathology and no evidence of spinal cord compression. Electromyography (EMG) revealed widespread active and chronic denervation, which suggested an anterior horn cell disorder or motor neurone disease (MND). However, the presence of sensory symptoms, constitutional features, and progressive clinical deterioration prompted further investigation. CT of the chest, abdomen, and pelvis identified cervical and retroperitoneal lymphadenopathy. Ultrasound-guided lymph node biopsy confirmed DLBCL. Cerebrospinal fluid (CSF) analysis did not demonstrate definitive evidence of central nervous system (CNS) lymphoma. After six cycles of polatuzumab vedotin, rituximab, cyclophosphamide, doxorubicin, and prednisolone (Pola-R-CHP), the patient achieved complete metabolic remission and showed substantial improvement in her neurological symptoms.

This case highlights the importance of maintaining a broad differential diagnosis in patients presenting with atypical neurological syndromes. DLBCL may rarely present with neurological features that mimic MND. Although a definitive paraneoplastic neurological syndrome (PNS) could not be established, a possible paraneoplastic neurological process was considered. Recognition of systemic red-flag features, such as weight loss and lymphadenopathy, was pivotal in reaching the correct diagnosis.

## Introduction

Diffuse large B-cell lymphoma (DLBCL) is the most common subtype of non-Hodgkin lymphoma (NHL), accounting for approximately 30-40% of cases worldwide [1]. It is an aggressive lymphoid malignancy characterised by the proliferation of mature B lymphocytes and predominantly affects older adults. Clinical presentation is heterogeneous and commonly includes rapidly enlarging lymphadenopathy, constitutional symptoms, and extranodal involvement. Despite its aggressive nature, DLBCL is potentially curable with modern immunochemotherapy regimens [2]. Neurological manifestations of lymphoma may occur through several mechanisms, including direct infiltration of the central or peripheral nervous system, leptomeningeal disease, neurolymphomatosis, vascular complications, metabolic disturbances, infections, treatment-related toxicity, or immune-mediated paraneoplastic neurological syndromes (PNS). Such presentations may mimic primary neurological disorders and can cause significant diagnostic uncertainty, which may delay diagnosis and treatment [3].

Paraneoplastic neurological syndromes are rare immune-mediated disorders that occur as remote effects of malignancy and are not caused by direct tumour invasion or metastasis. The 2021 PNS-Care criteria introduced a structured diagnostic framework that incorporates neurological phenotype, neuronal antibody status, the presence of associated cancer, and duration of follow-up, which allows classification as definite, probable, or possible PNS [4]. We describe an unusual case of DLBCL presenting with clinical and electrophysiological findings suggestive of an anterior horn cell disorder or motor neurone disease (MND). This represents a rare example of electromyography (EMG)-confirmed widespread denervation mimicking MND in a patient who was subsequently diagnosed with DLBCL and experienced neurological recovery following lymphoma-directed treatment. This case report highlights the importance of reconsidering an initial diagnosis when atypical neurological features and systemic red-flag symptoms are present.

## Case presentation

A 67-year-old woman presented with a three-week history of severe lower back pain radiating to both lower limbs. She had no prior history of neurological disease, neuromuscular disorders, or autoimmune illness. The pain was associated with paraesthesia affecting all four limbs, progressive weakness, faecal incontinence, dysphagia, and unintentional weight loss, which eventually left her bedbound. Four months before admission, she had sustained a back injury that was managed conservatively for presumed musculoskeletal pain. Prior to symptom onset, the patient had been independent in all activities of daily living and had no significant mobility limitations.

Neurological examination demonstrated asymmetric weakness affecting all four limbs, predominantly on the left side, along with left lower motor neurone-pattern facial weakness. Muscle strength was graded according to the Medical Research Council (MRC) scale [5]. Shoulder abduction and adduction were preserved bilaterally (5/5). Elbow flexion and extension were reduced to 3/5 on the left and 4/5 on the right. Hip flexion was 4/5 on the left and 5/5 on the right, while knee extension was 5/5 on the left and 4/5 on the right. Bilateral toe dorsiflexion was reduced to 4/5. Deep tendon reflexes were generally diminished. Bilateral ankle clonus was present, while plantar responses remained flexor bilaterally. No other cranial nerve abnormalities were documented.

Given concerns regarding spinal cord pathology, CT imaging of the head and MRI of the spine were performed. CT imaging demonstrated no acute intracranial abnormality (Figure 1). MRI of the spine demonstrated multiple minor degenerative disc bulges but no evidence of spinal cord compression, cauda equina syndrome, or other structural abnormalities that could adequately explain the patient’s symptoms (Figure 2).

**Figure 1:**
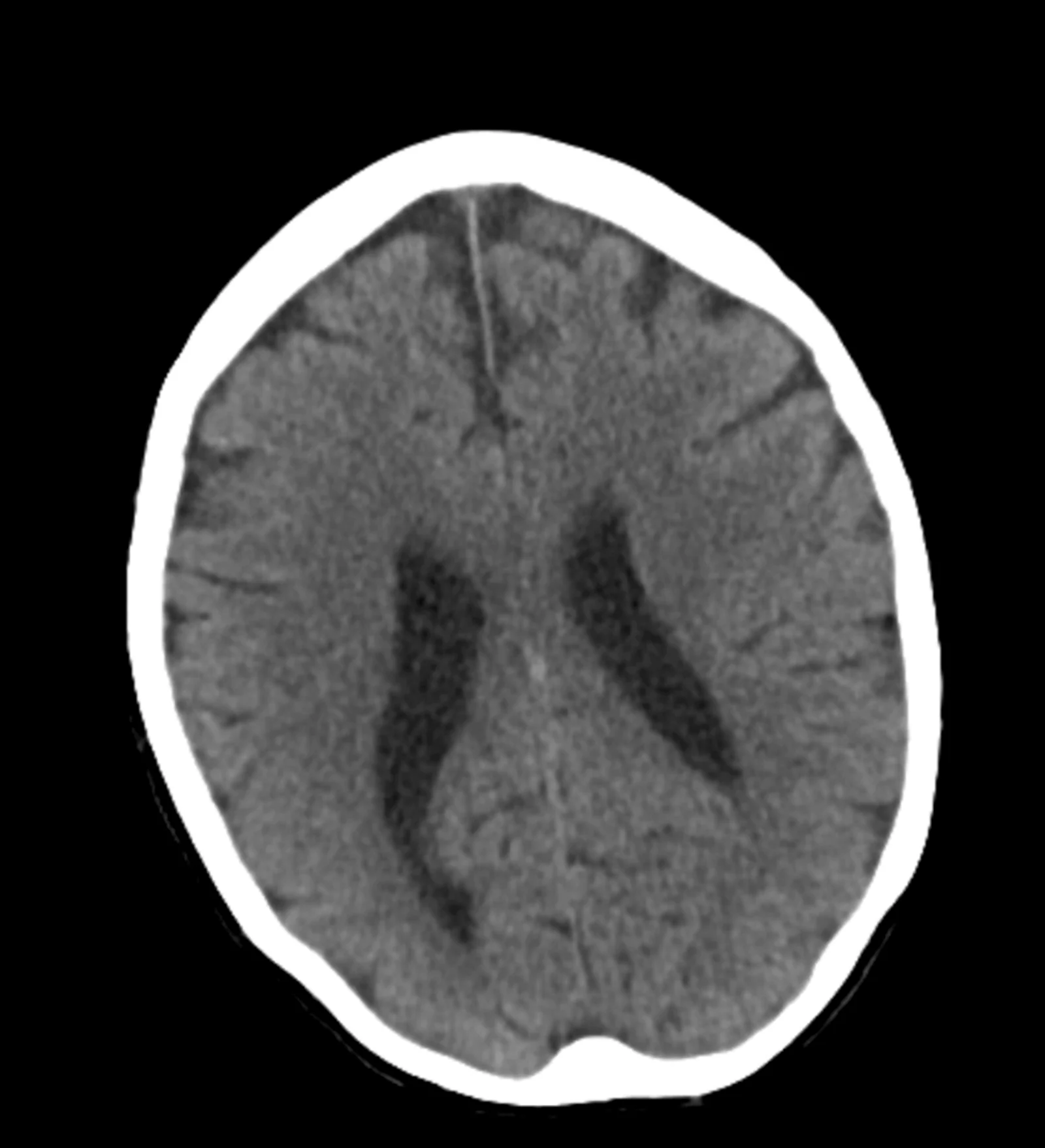
CT head showing no acute intracranial abnormality Non-contrast CT head demonstrates no acute intracranial abnormality. No intracranial haemorrhage, mass lesion, or acute ischaemic change was identified CT: computed tomography

**Figure 2:**
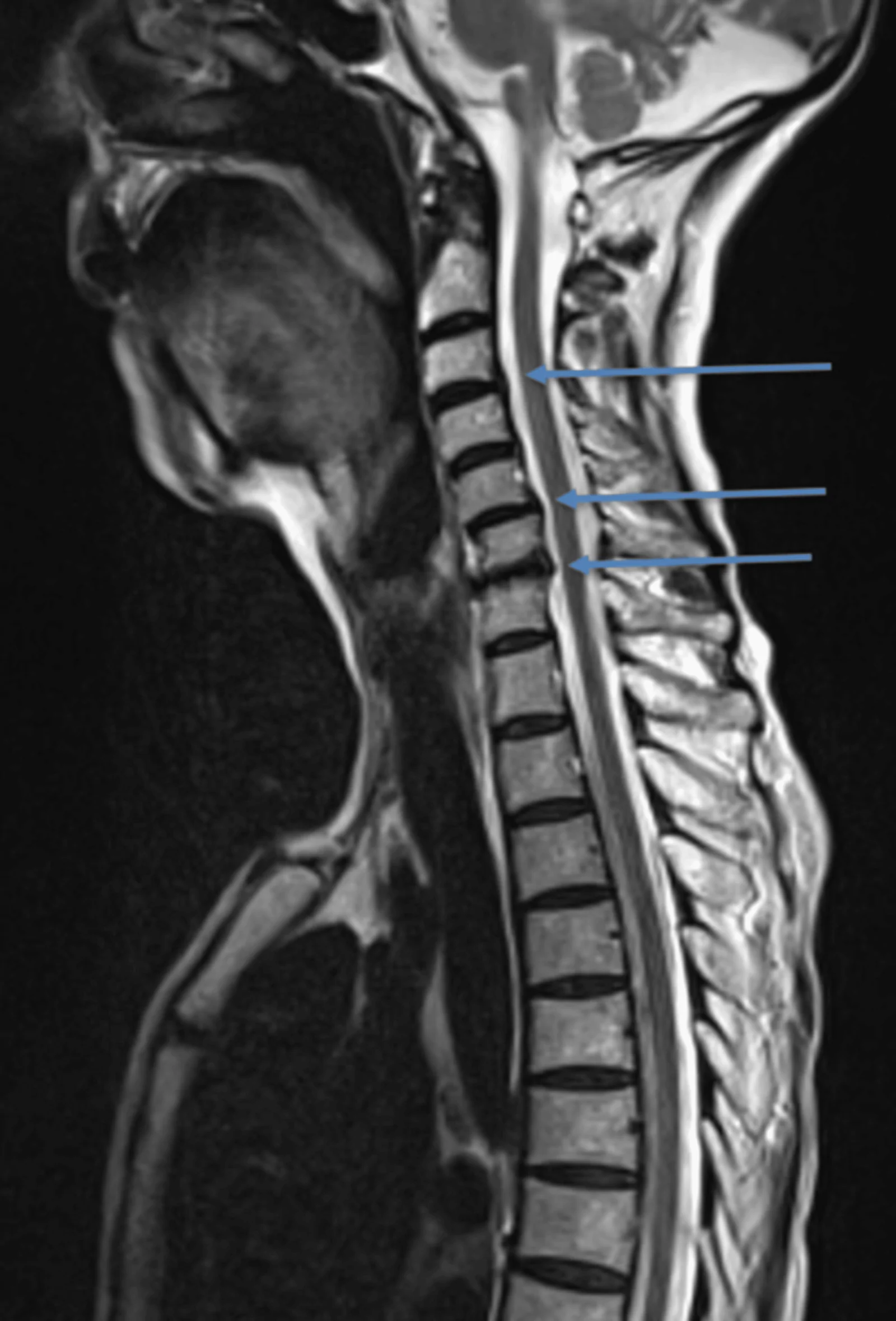
MRI cervical and thoracic spine demonstrating multilevel cervical disc disease MRI of the cervical and thoracic spine demonstrates multilevel cervical disc osteophyte complexes and disc bulges at C2-C3, C4-C5, and C5-C6, causing mild indentation of the thecal sac and mild right foraminal narrowing. No evidence of spinal cord compression or abnormal spinal cord signal was identified MRI: magnetic resonance imaging

As symptoms progressed, a brain MRI was performed, revealing chronic small-vessel ischaemic changes without evidence of acute infarction, demyelination, an intracranial mass lesion, or abnormal enhancement (Figure 3).

**Figure 3:**
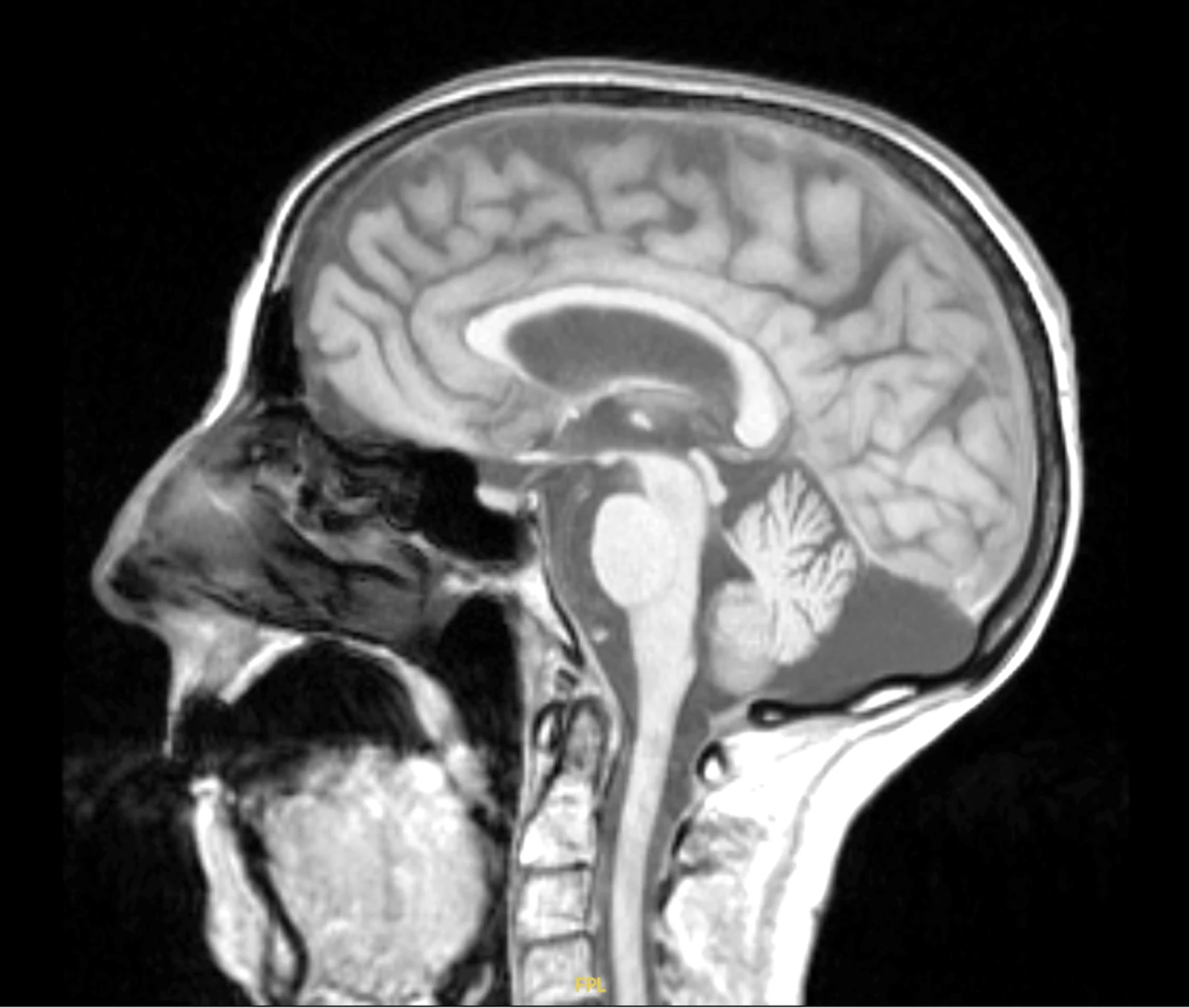
Post-contrast T1-weighted MRI brain Post-contrast T1-weighted MRI brain shows no abnormal intracranial enhancement. No focal intracranial lesion was identified; only chronic small-vessel ischaemic changes were noted MRI: magnetic resonance imaging

The neurosurgical team concluded that the spinal imaging findings were incidental and recommended conservative management. Pain was initially managed with oral morphine but subsequently required escalation to oxycodone, along with continuous subcutaneous midazolam, due to worsening symptoms. Formal neurophysiological assessment was subsequently performed on the recommendation of the neurology team.

Electromyography Findings 

Neurophysiological studies demonstrated preserved sensory conduction velocities and normal sensory responses (Figure 4). Motor responses involving the median, ulnar, tibial, and common peroneal nerves were significantly attenuated (Figure 5). EMG demonstrated widespread active and chronic denervation affecting the cervical, thoracic, lumbosacral, and bulbar segments. The neurophysiology report concluded: “This electrodiagnostic study shows significant electrophysiological evidence of widespread active and chronic denervation involving multiple spinal segments, including the cervical, lumbosacral, thoracic, and craniobulbar regions. These findings are suggestive of an underlying anterior horn cell disorder or motor neurone disease.”

**Figure 4:**
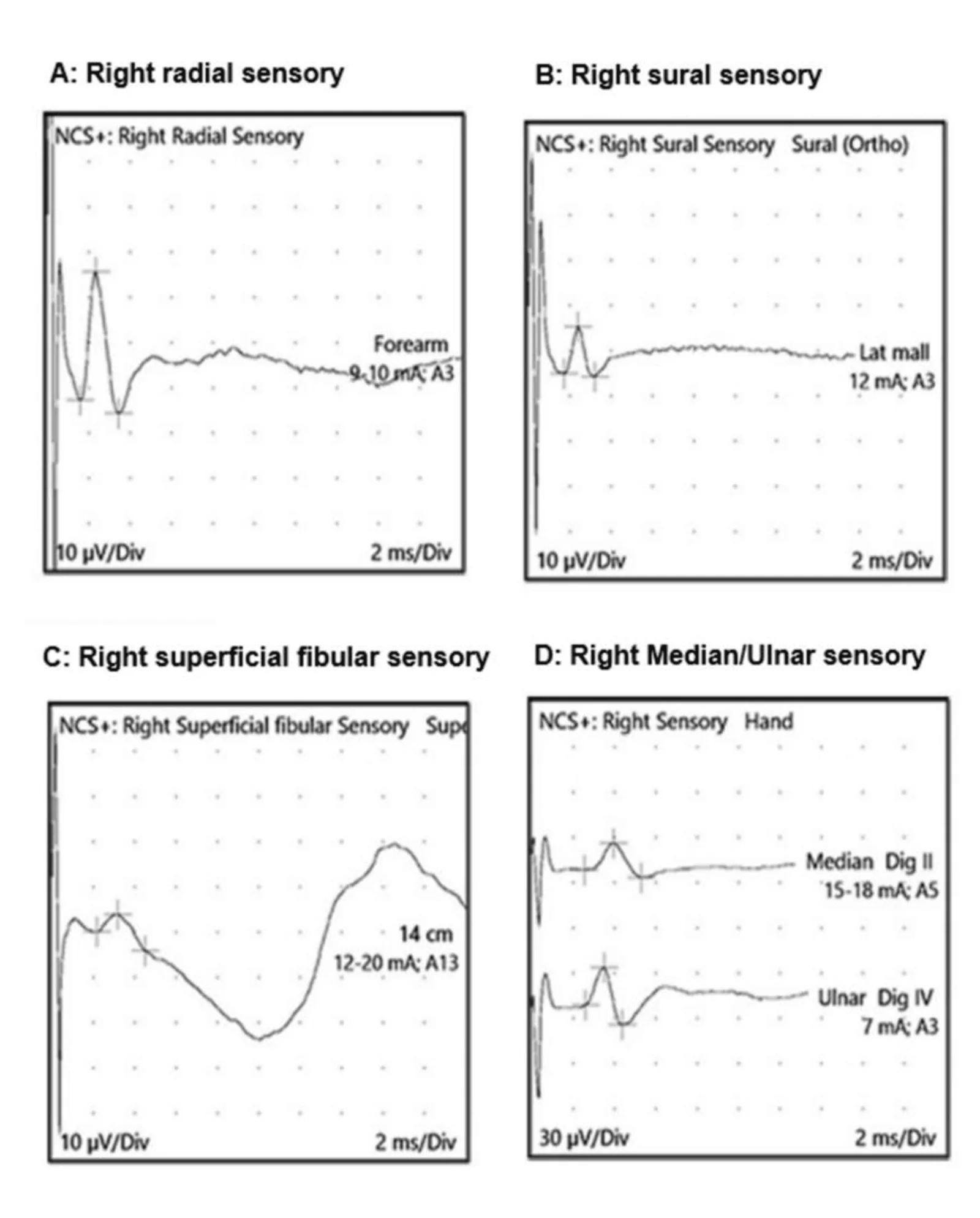
Representative sensory nerve conduction studies Sensory nerve conduction recordings from the right radial, sural, superficial fibular, median, and ulnar nerves demonstrate preserved sensory responses. Preservation of sensory nerve conduction favors a motor neurone disorder rather than a generalized sensorimotor neuropathy A. Right radial sensory. B. Right sural sensory. C. Right superficial fibular sensory. D. Right median/ulnar sensory

**Figure 5:**
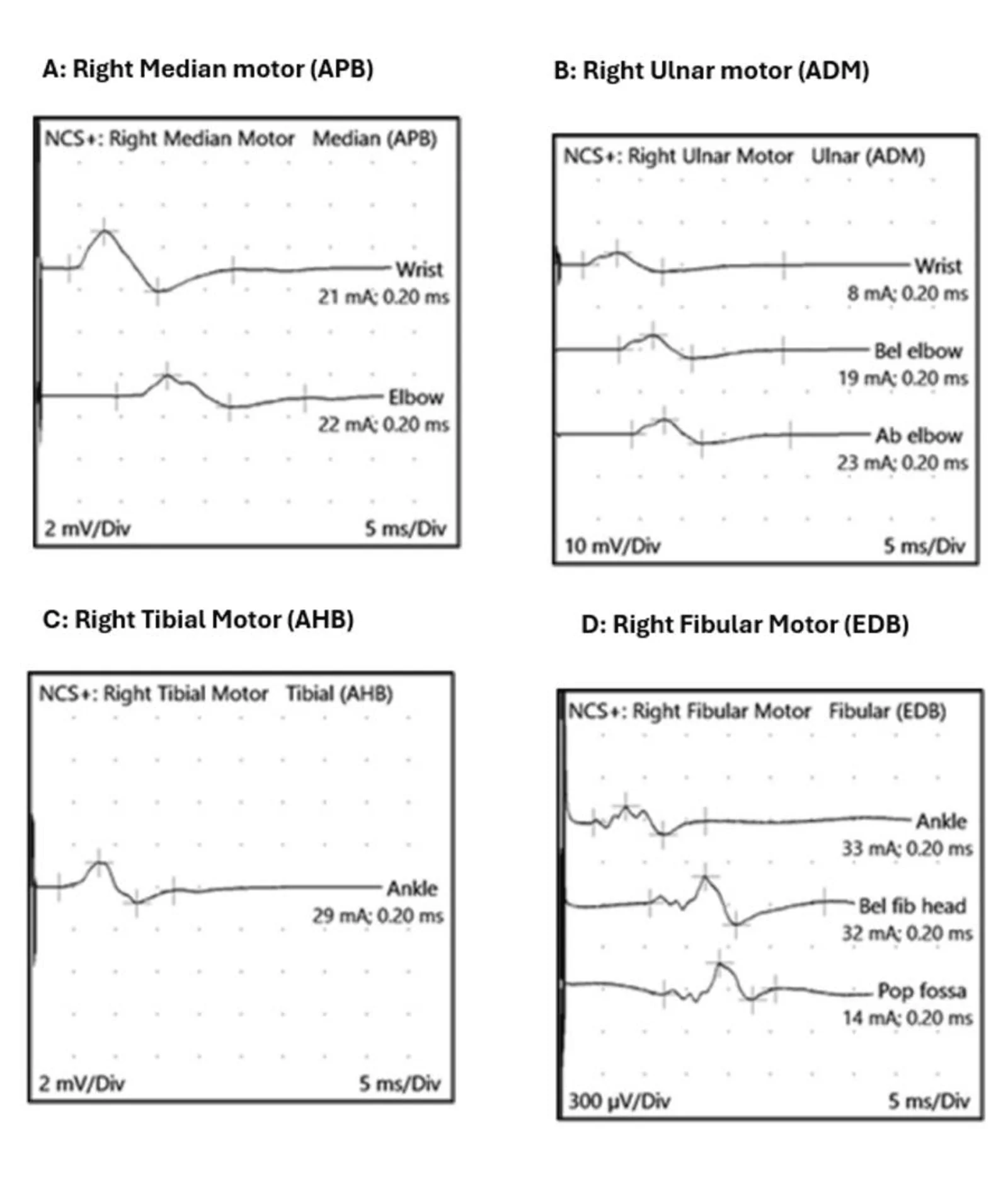
Representative motor nerve conduction studies Motor nerve conduction recordings from the right median, ulnar, tibial, and fibular nerves demonstrate reduced compound muscle action potential amplitudes, consistent with motor axonal involvement. Sensory responses remained preserved A. Right median motor (APB). B. Right ulnar motor (ADM). C. Right tibial motor (AHB). D. Right fibular motor (EDB)

Based on these findings, riluzole 50 mg twice daily was commenced. Despite the electrophysiological findings, the neurology team identified several atypical features that were inconsistent with classical MND, including sensory symptoms affecting all four limbs, bowel dysfunction, constitutional symptoms, and rapid functional decline. Consequently, alternative diagnoses continued to be explored.

During admission, cervical lymphadenopathy was identified on clinical examination. Given the patient's unexplained weight loss and progressive functional decline, contrast-enhanced CT imaging of the chest, abdomen, and pelvis was performed. This revealed extensive retroperitoneal and inguinal lymphadenopathy, raising suspicion of an underlying lymphoproliferative disorder (Figure 6).

**Figure 6:**
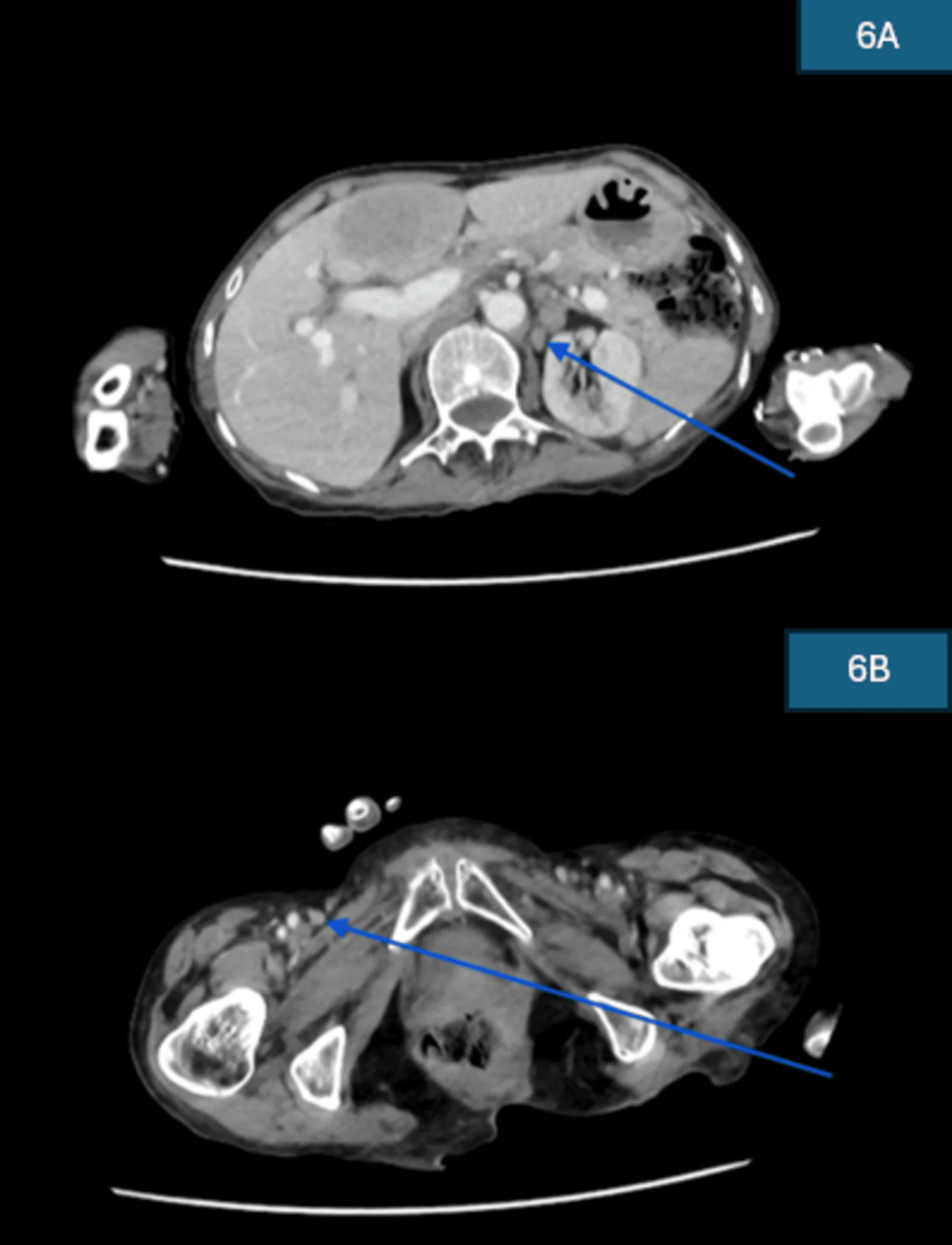
Contrast-enhanced CT chest, abdomen, and pelvis demonstrating lymphadenopathy suspicious for lymphoma (6A) Contrast-enhanced CT image demonstrates enlarged retroperitoneal lymphadenopathy (arrow). (6B) Contrast-enhanced CT image demonstrates enlarged iliac lymphadenopathy (arrow) CT: computed tomography

An ultrasound-guided lymph node biopsy was subsequently performed, confirming a diagnosis of DLBCL.

Lymph node biopsy report

Histopathological examination demonstrated sheets of large atypical lymphoid cells with numerous mitotic and apoptotic figures. Immunohistochemistry showed positivity for CD20, CD79a, PAX5, BCL2, BCL6, MUM1, and c-MYC, while CD3, CD10, and cyclin D1 were negative. The Ki-67 proliferative index exceeded 90%. These findings were diagnostic of DLBCL of non-germinal centre B-cell (non-GCB) phenotype.

Cerebrospinal fluid study

A lumbar puncture was performed to evaluate for central nervous system (CNS) involvement and exclude infectious causes. Cerebrospinal fluid (CSF) analysis demonstrated clear, colourless fluid with a white cell count of 19 cells/mm³ and a lymphocytic predominance (80%). Gram stain showed no organisms, and CSF culture demonstrated no growth. Polymerase chain reaction (PCR) testing for parechovirus, Epstein-Barr virus, enterovirus, adenovirus, herpes simplex virus, varicella-zoster virus, and cytomegalovirus was negative. Cytological examination was essentially acellular and showed no evidence of malignant cells. Overall, there was no definitive evidence of CNS lymphoma or CNS infection.

The case was reviewed at a multidisciplinary haematology meeting. Given the absence of a structural neurological explanation, the atypical neurological presentation, and the diagnosis of DLBCL, a possible paraneoplastic neurological process was considered. However, a definitive diagnosis of PNS could not be established because specific neuronal antibody testing was not performed and the available evidence did not fulfil contemporary PNS-Care criteria for definite PNS.

The patient underwent six cycles of polatuzumab vedotin, rituximab, cyclophosphamide, doxorubicin, and prednisolone (Pola-R-CHP). Following treatment, significant clinical improvement was observed. Appetite improved, pain decreased substantially, and neurological function also improved substantially. The patient is now able to mobilise with a walking stick and perform activities of daily living independently. Although residual weakness remained, the patient regained a considerably greater degree of independence than at diagnosis. End-of-treatment PET imaging demonstrated complete metabolic remission. The patient remains under regular haematology follow-up. The sequence of events from the patient's initial presentation through treatment and final clinical outcome is outlined in Table 1.

**Table 1:** Timeline of the clinical course Chronological summary of the patient's presentation, diagnostic workup, treatment, and clinical outcome CT: computed tomography; MRI: magnetic resonance imaging; EMG: electromyography; MND: motor neurone disease; CT CAP: computed tomography of the chest, abdomen, and pelvis; DLBCL: diffuse large B-cell lymphoma; MDT: multidisciplinary team; Pola-R-CHP: polatuzumab vedotin, rituximab, cyclophosphamide, doxorubicin, and prednisolone; PET: positron emission tomography

Timeline	Clinical event
4 months before admission	Back injury managed conservatively
3 weeks before admission	Progressive back pain and neurological symptoms developed
Admission	CT head and MRI spine performed
Early admission	Neurology and neurosurgical reviews
During admission	EMG suggestive of anterior horn cell disorder/MND
During admission	Riluzole commenced
During admission	Cervical lymphadenopathy identified
During admission	CT CAP demonstrated retroperitoneal lymphadenopathy
During admission	Lymph node biopsy confirmed DLBCL
Following diagnosis	Haematology MDT discussion
Months 1-6	Six cycles of Pola-R-CHP administered
End of treatment	PET demonstrated complete metabolic remission
Follow-up	Significant neurological improvement

## Discussion

This case report illustrates the diagnostic complexity that may arise when DLBCL presents predominantly with neurological manifestations. DLBCL is characterized by marked clinical heterogeneity and most commonly presents with lymphadenopathy, constitutional symptoms, or extranodal disease [1,2]. Neurological symptoms are relatively uncommon as initial presenting features and may obscure the underlying diagnosis. The differential diagnosis initially included spinal cord compression, cerebrovascular disease, MND, neurolymphomatosis, inflammatory neuropathy, CNS lymphoma, and paraneoplastic neurological syndromes. Structural causes were effectively excluded through neuroimaging, while electrophysiological findings guided further investigation towards an anterior horn cell disorder.

Amyotrophic lateral sclerosis (ALS), the most common form of MND, is characterized by progressive degeneration of upper and lower motor neurones, producing weakness, muscle wasting, fasciculations, and reflex abnormalities. Although the EMG findings in our patient were highly suggestive of MND, the overall clinical picture was atypical. Prominent sensory symptoms, bowel dysfunction, constitutional symptoms, and rapid progression are not characteristic features of classical ALS and should prompt consideration of alternative diagnoses [6,7]. The identification of widespread lymphadenopathy ultimately led to a tissue diagnosis of DLBCL and substantially changed the management approach. This underscores the importance of maintaining a broad differential diagnosis when evaluating patients with presumed MND, particularly when systemic red flags are present.

Neurological complications associated with lymphoma may occur through multiple mechanisms, including direct tumour infiltration, leptomeningeal disease, neurolymphomatosis, vascular injury, metabolic abnormalities, and paraneoplastic autoimmune phenomena [8]. Neurolymphomatosis was considered in this case because it may present with painful neuropathy, weakness, and multifocal neurological deficits. However, MRI did not demonstrate overt nerve infiltration, and CSF analysis showed no malignant cells. Nevertheless, neurolymphomatosis cannot be completely excluded because MRI and CSF cytology lack sensitivity, and neither dedicated FDG-PET assessment of peripheral nerves nor nerve biopsy was performed [9,10,11].

CSF examination demonstrated mild lymphocytic pleocytosis but no microbiological evidence of infection or cytological evidence of lymphoma. Negative CSF cytology alone does not completely exclude CNS lymphoma, as its sensitivity remains limited, particularly in early disease [12,13]. A paraneoplastic neurological mechanism was also considered. According to the updated PNS-Care criteria, diagnosis relies upon the integration of the neurological phenotype, neuronal antibody status, associated malignancy, and clinical follow-up [14]. In this patient, neuronal antibody testing was unavailable, and therefore the available evidence was insufficient to establish a diagnosis of definite or probable PNS according to contemporary diagnostic criteria.

In addition to neurolymphomatosis, the differential diagnosis included a broader lymphoma-associated immune-mediated neurological syndrome. Features supporting this consideration included asymmetric tetraparesis, lower motor neurone facial weakness, sensory disturbance, bowel dysfunction, bilateral ankle clonus, widespread active denervation on electromyography, and mild CSF lymphocytic pleocytosis. However, the available findings were insufficient to establish a diagnosis of paraneoplastic encephalomyelitis or another definite paraneoplastic neurological syndrome according to PNS-Care criteria. Furthermore, incomplete neuronal antibody testing, the absence of pathological confirmation, unremarkable conventional neuroimaging, and the inability to definitively exclude neurolymphomatosis all limit diagnostic certainty. Therefore, any lymphoma-associated immune-mediated neurological mechanism remains speculative.

Nonetheless, several observations support a possible association between the neurological syndrome and the underlying lymphoma. There was no convincing structural explanation for the neurological deficits; symptoms developed in temporal association with newly diagnosed lymphoma, and substantial neurological recovery occurred following successful lymphoma-directed treatment. Clinically, the patient progressed from being bedbound at presentation to mobilising with a walking stick and independently performing activities of daily living following completion of therapy. Although detailed follow-up neurological examination findings, including formal strength grading, reflex assessment, and sensory evaluation, were unavailable, the marked functional recovery would be unusual in classical ALS and supports a relationship between the neurological syndrome and the underlying malignancy [15].

This case report reinforces the importance of revisiting initial diagnostic assumptions when clinical findings fail to fit a typical disease pattern. Recognition of sensory disturbance, bowel dysfunction, constitutional symptoms, lymphadenopathy, and rapid clinical deterioration ultimately led to the diagnosis of a potentially curable haematological malignancy.

Limitations

This report has several limitations. Comprehensive paraneoplastic antibody testing was not performed, which prevented definitive classification according to PNS-Care criteria. Delayed CSF processing limited further immunophenotypic analysis, and neurolymphomatosis could not be definitively excluded because dedicated peripheral nerve imaging and nerve biopsy were not undertaken. Baseline PET-CT imaging was unavailable, restricting assessment of the pre-treatment metabolic disease burden. In addition, representative histopathological images from the diagnostic lymph node biopsy could not be retrieved despite repeated attempts. However, the diagnosis of DLBCL was confirmed by formal histopathological and immunohistochemical assessment. Therefore, while the marked neurological improvement following lymphoma-directed therapy supports a lymphoma-associated neurological process, the precise underlying pathophysiological mechanism remains uncertain.

## Conclusions

This case report underscores the diagnostic challenges posed by atypical presentations of DLBCL, particularly when neurological manifestations mimic primary neuromuscular or neurodegenerative disorders. The patient’s presentation initially suggested an anterior horn cell disorder or MND, supported by electrophysiological findings of widespread denervation. However, the presence of sensory symptoms, bowel dysfunction, constitutional features, and progressive systemic decline prompted further investigation, ultimately revealing an underlying aggressive lymphoma. This report highlights the need to investigate for underlying malignancy when systemic red-flag features, particularly unexplained weight loss and lymphadenopathy, are present in patients with presumed MND.

Although a definitive diagnosis of PNS could not be established, a possible paraneoplastic neurological process was considered after alternative explanations had been investigated. However, the available evidence did not fulfil contemporary PNS-Care criteria for definite or probable PNS, and the precise mechanism underlying the neurological presentation remains uncertain. This report also highlights that electrophysiological findings suggestive of an anterior horn cell disorder or MND should be interpreted within the broader clinical context, as lymphoma-associated neurological syndromes may produce misleading EMG findings and mimic primary neurodegenerative disease. Furthermore, multidisciplinary collaboration involving neurology, neurosurgery, radiology, pathology, and haematology was essential to challenge the initial diagnostic impression, facilitate further investigation, and ultimately establish the correct diagnosis. The report emphasises the importance of maintaining a broad differential diagnosis, recognising systemic red-flag features, and reconsidering initial diagnostic assumptions when clinical findings are atypical. Successful treatment of the lymphoma resulted in complete metabolic remission and significant neurological improvement, highlighting the importance of timely tissue diagnosis in patients with unexplained neurological syndromes.
